# Rokkan Rules? Communist Elites and the Choice of Electoral Systems in the Yugoslav Republics, 1989–1990

**DOI:** 10.1080/09668136.2023.2242604

**Published:** 2023-08-24

**Authors:** Josip Glaurdić, Ensar Muharemović

## Abstract

We use the previously neglected cases of the Yugoslav republics to revisit the question of how electoral systems were formed for the first elections during the transition from communism in 1989–1990. By exploring archival and other sources created contemporaneously by the relevant decision-makers, we build on Rokkanian interpretations of electoral system design. Unlike Rokkan, however, we do not see parties as unitary or united actors. Our analysis instead focuses on the leadership and the dominant wings of the ruling parties and shows that their preferences regarding electoral rules served their intra-party ambitions and reflected their intra-party power capacities.

Comparative studies of the decision-making of communist elites in the creation of democratic electoral systems in postcommunist Eastern Europe largely neglect the cases of the Yugoslav republics: Bosnia–Hercegovina, Croatia, Macedonia, Montenegro, Serbia and Slovenia.^1^ This is a serious shortcoming of the literature, since elections in the Yugoslav republics were arguably the most consequential in all of Eastern Europe: in addition to marking the advent of multipartyism, they brought to power politicians who precipitated the federation’s descent into dissolution and bloody wars. Explanations based on the fact that these were merely regional elections (Linz & Stepan 1992) are not convincing because Yugoslav republics were constitutionally defined as sovereign states in a very decentralised federation; they had their own communist parties, nascent and unique opposition forces, and distinctive paths to democratisation. Moreover, since no elections on the federal level were ever held, these elections served as the true markers of the republics’ transition to multiparty democracy, however flawed. The Yugoslav brand of communism/socialism had its unique characteristics, given the federation’s 1948 split with Moscow.^2^ For all the liberties Yugoslavia offered, however, the power logic of its ruling system and the nature of its transition to multiparty democracy had much in common with similar processes elsewhere in Eastern Europe. The neglect of Yugoslavia’s six republics in comparative studies of the early stages of transitions to democracy and institutional development is particularly regrettable because Yugoslav republics are ideal subjects for comparative study due to their economic, cultural, political and social similarities, as well as their institutional uniformity prior to the fall of communism and the vastly different institutional choices made by the communist elites during the crucial 1989–1990 period. Without understanding those founding institutional choices, we cannot fully understand the nature of the later stages of democratic transition and the origins of electoral systems not only in Yugoslavia’s successor states but also throughout postcommunist Eastern Europe.

This article remedies the literature’s shortcoming in this respect. Our approach is rooted in the belief that we can elucidate institutional choices made by policymakers only by ‘reading history forward’—by immersing ourselves in the sources created simultaneously with the political decisions we aim to explain (Ahmed 2010). Using internal documents of the ruling communist parties, parliamentary debates and expert opinions uncovered in local archives,^3^ as well as contemporaneous press reports, diaries and public interviews with the relevant decision-makers, we reveal how three very different electoral systems were chosen by the communist elites in Croatia, Bosnia–Hercegovina and Serbia during 1989 and 1990. We focus our comparative case studies on the communist elites of these three republics for three reasons: they were the largest republics in the federation; the results of their elections were the most important for the country’s survival; and, most importantly, their diverse political situations represented three different levels of balance between the forces under the respective communist leadership’s control, on the one hand, and the nascent opposition forces, on the other, a balance that we believe was the crucial deciding factor in the choice of electoral systems at the time.

Our narrative builds on the politically rationalist interpretations of the choices of electoral systems in West European democracies at the time of the expansion of suffrage more than a century ago, the so-called first wave of democratisation, rooted in the scholarship of Stein Rokkan (Rokkan 1970; Boix 1999, 2010; Leemann & Mares 2014). We also build on the studies of electoral system design in postcommunist Eastern Europe that likewise trace institutional design to politically rationalist actors operating under conditions of uncertainty (Lijphart 1992; Benoit & Schiemann 2001; Benoit & Hayden 2004). We agree with those who see the choice of electoral system as a primarily political decision defined by calculations about access to power. Our set of propositions, however, offers two important caveats to this school of thought that we believe need to be integrated into the literature on electoral system design, particularly in the literature on the third wave of democratisation that began in the 1970s and includes the postcommunist transition of the 1990s.

First, unlike virtually all scholarship in the Rokkanian tradition, we do not see political parties as unitary actors. Instead, we unpack them and focus on actors who had real decision-making power in the Yugoslav republics: leaderships and the dominant wings of the ruling communist parties. The level of intra-party (or, rather, intra-system) unity is actually one of the critical factors we see as determining party leaders’ strategies. Our narrative recognises the messiness of transitional politics and the fact that ruling regimes were often aggregates of different factions. Second, and also in contrast to other scholarship in the Rokkanian tradition, we see power the same way ruling regime leaders saw it when they were defining their preferences over electoral rules, that is, more broadly than their parties’ potential to win seats. For us, as well as for them, their intra-party power mattered just as much as their parties’ projected electoral strength. Preferences over electoral rules were thus formed with both of these dimensions in mind. Seat maximisation was obviously crucial, but it had to serve the power goals (and reflect the power capacities) of the party leadership.

Aside from these two necessary caveats, the explanation for the adoption of electoral rules in the Yugoslav republics presented in this article follows a Rokkanian logic. Where the communist power-holders controlled their own parties and the left end of the spectrum while facing a weaker and divided centrist/right-wing opposition, they opted for majoritarian rules, as was the case in Serbia. Where the communist leaders had a weak grip over a divided front of system parties and/or they faced an organised challenge on the left end of the spectrum, which was unwilling to engage in electoral coordination, while facing a strong and united centrist/right-wing opposition, they opted for PR rules of high proportionality. This was the case in Bosnia–Hercegovina. Finally, where the balance of power was uncertain—either because system power-holders had limited control over their own parties or because they faced a leftist challenge, while the strength of the divided centrist/right-wing opposition was unclear—they opted for variations on the ideal types of electoral rules that were supposed to provide them with strategic intra-party or electoral benefits. In Croatia, these were the French-style double-ballot rules with low thresholds for entry into the second round, designed to serve as primaries to first placate and then ultimately defeat the disobedient wings of the ruling regime.

Our analysis makes several important contributions to understanding the choice of electoral systems by ruling elites during the third wave of democratisation. It explains a set of significant and previously neglected cases using rigorous historical research and primary sources. It also tests and ultimately demonstrates the value of classical theoretical approaches in explaining electoral reforms during the first wave of democratisation to our broader understanding of the early transition period, while at the same time highlighting refinements needed to bring theory closer in line with political practice.

## Ruling elites and electoral system formation: theoretical and empirical approaches

The study of the origins of electoral systems over the past half a century has been dominated by Stein Rokkan’s (1970) seminal analysis of electoral system formation in West European democracies. Rokkan’s principal proposition, building on earlier work by Karl Braunias (1932), was that proportional representation in the early stages of West European democracies was adopted by ruling parties where increasing competition led either to potentially destabilising levels of disproportionality of political minorities in culturally diverse polities or to the ruling right-wing parties feeling threatened by the expansion of suffrage and the resulting rise of left-wing parties representing the working class. Rokkan’s second proposition understandably garnered far more interest in the field because it fit the popular understanding of the political machinations taking place during the period, and because it was an elegant argument that could be easily understood and formalised.

Carles Boix’s (1999) influential study breathed new life into Rokkan’s proposition about this second path to proportional representation and sparked a lively debate over the past two decades that is unlikely to be settled. Boix preserved Rokkan’s straightforward narrative of the choice of electoral systems being primarily a political question of access to power in an electoral arena where players are political parties as unitary actors. For Boix, the key aspects of the story concerned the perceived balance of power between the parties in power and the ascendant challengers, and the capacity of the parties in power to coordinate their responses to the changing situation. The proportionality of electoral rules was thus increased (namely, there was a shift from majority/plurality rules to PR) when the left-wing challengers were strong and the right-wing incumbents were weak and/or unable to coordinate a common response. If the right-wing incumbents were strong and/or able to create a coordinated response to the challengers, electoral rules remained in their majority/plurality *status quo*.

This political story of the origins of electoral systems, however, has not been uncontested. Setting aside the challenge presented by the political economists who have tried to relate countries’ electoral institutions to the nature of their economies (Rogowski 1987; Cusack *et al*. 2007), the Rokkan/Boix story has been put under the magnifying glass by a number of comparative studies with firmer grounding in historical research. Marcus Kreuzer’s (2010) replication of Boix’s findings demonstrated that the general story was robust to different specifications, and Boix’s (2010) own refinement of his original argument provided evidence that it was exactly the parties that were the most threatened by the ascendant left-wing challengers that supported the institution of proportional representation, as one would expect, based on his original argument. The story that comes through a number of other in-depth accounts of decision-making during crucial episodes of building or altering of electoral institutions in the first wave of democratisation, however, is one of greater complexity.

Similar to Boix, Leemann and Mares (2014) found that the implementation of proportional representation in 1912 Germany was indeed supported by politicians most threatened by the ascendant social democrats but also by those faced with greater disproportionality regardless of the social democratic threat. In other words, they found that Rokkan’s two paths toward PR worked in tandem in the crucial German case. Other studies offered a more fundamental challenge to the Rokkan/Boix story by showing that parties could not be understood as unitary actors in pursuit of seat maximisation. Amel Ahmed (2010) in her comparative study of Belgian and British electoral system choices showed, first, that contention over electoral rules did not only happen among but also within parties and, second, that choices over proportional or majoritarian electoral rules cannot be properly understood outside of parties’ and politicians’ more general preferences regarding the nature of the process of democratisation. Cox *et al*.’s (2018) study of the introduction of proportional representation in Norway after World War I similarly showed that the choice of electoral system was driven by the nature of intra-party conflicts and demonstrated that proportional representation was the preferred option of party leaders. Party leaders wanted to increase party cohesion and internal control, and having centralised nomination practices under PR helped them achieve that. Schröder and Manow (2020) demonstrated that the same intra-party dynamics were crucial in the reforms leading to greater proportionality in Germany in the early twentieth century.

We draw two general lessons from this more recent scholarship on electoral system formation in the early decades of West European democracies. First, that political conflicts over institution-building cuts not only between but also within parties, making the focus on the intra-party dimension of competition interesting and important. Second, that the goals of those able to effect institutional change were related to political power understood more broadly than their parties’ ability to win seats; that is, under certain conditions, intra-party power could be as valuable as inter-party power.

These two lessons have been largely absent from the literature on electoral system design during the third wave of democratisation in Eastern Europe. Early analyses of electoral system design during postcommunist transitions to democracy virtually all adopted the framework of parties as unitary actors pursuing seat maximisation (Lijphart 1992; Geddes 1996; Elster *et al*. 1998). From the Hungarian roundtable negotiations over electoral rules (Benoit & Schiemann 2001), through debates on the early democratic electoral laws in Russia (Remington & Smith 1996), to electoral system malleability in 1990s Poland (Benoit & Hayden 2004), the narrative of East European electoral system designers during the early stages of transition to democracy has been one of myopic political parties interested only in improving their own electoral fortunes. In some instances, some (mainly democratic opposition) parties may have been concerned with larger issues, such as the character of democracy they were building (Renwick 2005) or emulating pre-communist institutions (Benoit 2007). Nevertheless, the general picture of the process of design of foundational electoral systems in Eastern Europe (particularly with regard to the ruling communist parties) in the first two decades of scholarship on this period remains rather uniform. Parties were unitary actors, primarily interested in seat maximisation, though with one crucial difference compared to West European parties during the first wave of democratisation: uncertainty.^4^ Unlike West European electoral system designers of a century ago who had the benefit of learning through at least some (quasi-democratic) electoral competition prior to the expansion of suffrage, East European parties operated in a much more fluid environment with supposedly unclear electorate loyalties. They were thus much more prone to make colossal mistakes, as the Polish communists famously did in choosing majoritarian electoral rules for contested seats in the partially free 1989 elections (Kaminski 2002).

The literature’s focus on parties’ electoral calculus as the decisive factor in their decision-making with regard to electoral system design is perfectly understandable, especially in the context of transition from communism where the ruling parties were facing a potential deluge that could not only sweep them out of office for one term but also block them from public life in perpetuity. However, conceptualising the ruling communist parties as unitary actors and not recognising the intra-party dimension of political competition at the time does not reflect reality. The ruling communist parties were in flux throughout Eastern Europe. In some countries, they did remain monolithic; in most cases, they were riven by internal conflict. These conflicts were usually between some variety of reformers/liberals and dogmatists/conservatives, though in a number of places they were also defined along ethnic lines. We believe the ruling parties’ internal unity, together with the nature of development of the nascent opposition, played a critical part in the institutional choices of those who had the power to determine parties’ policy positions. Crucially, these players—most often found in the positions of authority in party central committees—effected policy with their own goals and power in mind that at times went beyond seat maximisation for their parties.

Over the past decade or so, there have been few new studies of foundational electoral systems during transition from communism in Eastern Europe. In one such excellent study, Nina Barzachka (2014) has shown that we do need to expand our view of how ruling communist elites perceived power in their decision-making on electoral system design. She showed that Bulgarian communists/socialists were willing to compromise with the opposition and agreed to a system that would result in a ‘tactical loss’ of some seats but would also ensure greater legitimacy of their projected victory and goodwill, which could be useful in the later process of transition. We find great value in this interpretation because it forces us to expand our view of political power beyond short-term seat maximisation. Nevertheless, we deem the portability of its conclusions limited due to the nature of the case under scrutiny.

Our focus is instead on how the communist elites perceived the balance between their intra- and inter-party strength. We believe it mattered whether communist leaders had a firm grip over the whole party apparatus or whether they presided over a divided and/or disintegrating structure of competing wings and platforms, and that it also mattered whether they faced a weak and divided opposition that had limited chances for electoral success or a united and strong opposition front determined to overthrow them. We suggest that the communist leaders’ electoral system preferences generally reflected this balance of power in a rather straightforward Rokkanian fashion: intra-party weakness and divisions paired with opposition strength and unity led to communist leaders’ preference for proportional rules. The reverse led to their preference for majoritarian rules. Where this balance of power was unclear, however, communist leaders devised electoral rules that reflected their potentially conflicting goals of dealing with intra-party/intra-system challengers and maximising party seats in an electoral competition with the opposition. Throughout that process, they demonstrated a capacity to learn and innovate because they had a reasonably sound understanding of the effects different electoral system provisions would have on the projected electoral results and the nature of intra- and inter-party competition, regardless of the uncertainty inherent in the historical context of that time.

## Setting the stage: Yugoslav electoral institutions and the advent of democracy

The decade preceding the first democratic elections in the Yugoslav republics was a time of economic, social and political upheaval. The death of Tito in 1980 marked Yugoslavia’s descent into crisis that steadily ate away at the legitimacy of the communist regime. Yugoslavia was a strongly decentralised federation of six republics and two autonomous provinces within Serbia: Vojvodina and Kosovo. Although it was ostensibly ruled by one communist party—officially known as the League of Communists of Yugoslavia (*Savez komunista Jugoslavije*—SKJ)—the federation actually had a complex system of representation and government. Yugoslavia had nine Leagues of Communists—one for each republic and autonomous province, as well as for the Yugoslav People’s Army (*Jugoslavenska narodna armija*—JNA)—and all these parties had different ideas how to answer not only the mounting economic problems such as hyperinflation, falling incomes, rising unemployment, international debt and chronic shortages of basic supplies, but also how to respond to the popular challenges to the legitimacy of their rule.

Prior to the first democratic elections held in 1990, Yugoslavia did not have experience with true democracy. In the interwar Kingdom of Yugoslavia, elections were sham affairs marred by political violence and blatant vote-rigging by the parties loyal to the regime (Kasapović 2014). For example, after the 1931 elections where voters were allowed to vote only for the list supporting the royal dictatorship, elections were run under rules inspired by the electoral system instituted by the Italian fascists, bringing massive bonus seats to the parties in power in order to ensure their undisputed grip on policymaking (Balkovec 2017). Unlike some other Eastern European countries, such as Czechoslovakia or Bulgaria (Benoit 2007), Yugoslav republics had no interwar electoral tradition to fall back on.

Communist elections after their victory in World War II also offered voters no real choice and served as mobilising rituals (Spehnjak 1991). After the 1974 constitutional changes that strengthened the country’s decentralisation to the republics and autonomous provinces and instituted the so-called ‘delegate system’, voters could only elect their representatives indirectly, in the tricameral republican parliaments consisting of the Socio-Political Chamber (lowest house), Chamber of Municipalities and Chamber of Associated Labour (Grdešić *et al*. 1986). Parliamentarians were chosen among and by the voters’ ‘delegates’ in the tricameral councils of local municipalities. These municipal delegates were the only directly elected representatives, and they were elected *via* candidate lists for the lowest municipal chamber and in single-member districts with plurality rules for the two remaining municipal chambers. Crucially, however, elections were preceded by an arduous nomination and candidate approval process in so-called ‘people’s assemblies’, which had to be conducted through the Socialist Alliance of Working People (*Socijalistički savez radnog naroda*—SSRN), the successor organisation of the World War II Popular Front. This process was essentially directed by the republican Leagues of Communists and their local activists. Unsurprisingly, such a system of representation led to widespread apathy and criticism in spite of artificially inflated turnout figures. This was broadly recognised throughout the country, leading to the 1988 changes in the federal constitution mandating direct elections of representatives to republican and federal parliaments, albeit with the communist-dominated rules of candidate nomination and approval left intact (Sokol 1988).

Faced by the need to bring their republican constitutions and electoral laws in line with the changes to the federal constitution, Yugoslav communists were forced to make important choices regarding setting the rules of the game for the upcoming elections in which they may have had to face real opposition. Their differences became more pronounced throughout 1989, culminating in two opposing approaches taken by the communist leaderships of Slovenia and Serbia that autumn and winter. Although the clash between Slovenian and Serbian camps was dominated by questions of federalism, constitutional reforms and rising nationalism, in its essence it was also a clash of diametrically opposing views of democratisation, representation and electoral competition. Whereas Slovenian communists were for increased decentralisation and liberalisation, the Serbian leadership under Slobodan Milošević was for the recentralisation of the federation and more limited democratisation through so-called non-partisan representation that would essentially continue to be dominated by the communists.

These two approaches clashed at the January 1990 Congress of the League of Communists of Yugoslavia. Virtually all amendments to the federal party platform by the Slovenian delegation at the Congress were rejected by the more numerous Serbian delegates and their allies. The Slovenian delegation walked out, closely followed by the bulk of the Croatian delegation. Although the rump congress continued its work later that May, the January congress marked the end of the federal League of Communists. Republican communist organisations continued on their individual paths of internal reforms and building electoral institutions, with very different results and levels of electoral success.

The diversity of the chosen systems of representation was very broad, even though in each republic the process was determined with little input from the emerging opposition. Out of the six League of Communists leaderships, only two chose the same electoral system for the lowest/single house of the new republican parliaments: Croatian and Macedonian communist leaderships opted for the French-style two-round majority/plurality rules with a 7% threshold for entry into the second round. Slovenian, Bosnian and Montenegrin communist leaderships opted for proportional representation rules, although with vast differences in parliamentary size, average district magnitude,^5^ allocation formula and threshold, while the Serbian communist leadership opted for simple two-round majoritarian elections in single-member districts.

**FIGURE 1.**
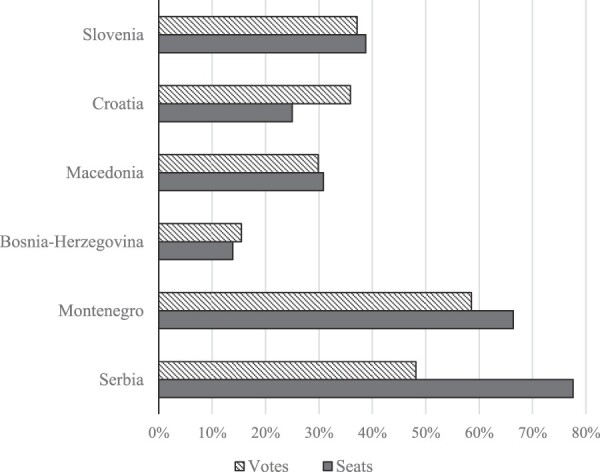
Votes and Seats Won by System Parties in the 1990 Elections

The results communist parties and their allies managed to achieve in the six electoral competitions throughout 1990, shown in Figure 1, were as different as the sets of electoral rules they chose. In the spring elections in Slovenia and Croatia, the establishment parties suffered clear defeats, though they did much better than their Hungarian and Czechoslovak counterparts, which earned only about 15% of the votes around the same time. The communist defeat was particularly painful in Bosnia–Hercegovina where the nationalist parties representing the republic’s Muslims, Serbs and Croats swept to power. Ultimately, the regime parties won only in Montenegro and Serbia, reaping the benefits of electoral system disproportionality through clever institutional design and correct assessment of the balance of electoral forces.

## Croatia: managing internal divisions with French-style two-round elections

Throughout 1988 and 1989, the leadership of the Croatian League of Communists (*Savez komunista Hrvatske*—SKH) became internally split between hardliners (so-called ‘dogmatists’) and liberals, and the membership of the party became split between the supporters and opponents of Serbia’s platform for the recentralisation of the Yugoslav federation. The problem was compounded by the fact that this internal cleavage coincided with ethnic identification. Croatian Serbs were disproportionately represented in the party membership: they constituted 11% of Croatia’s population and 25% of SKH membership. According to the secretary of the SKH Central Committee Presidency, Drago Dimitrović, Croatian Serb communists found much to like in the platform of the Serbian regime of Slobodan Milošević and pressured the Croatian party to fall in line behind Serbia’s leader, while threatening to create a splinter organisation of their own if this was not done (Lovrić 1989a). The SKH leadership was also losing popular legitimacy among the majority Croat population because it did not stand up to Milošević. Public opinion polls found all communist functionaries deeply unpopular, with the electoral appeal of those from the dogmatist wing in low single digits.^6^ The electorate was, however, adrift because it did not have anyone to support (Jović 1989). The emerging Croatian opposition was so weak and divided that one of its leaders claimed they would all ‘fit into two police vans’ (Babić 1989). Communist leaders seemed to agree: at a private social event of the communist liberal leaders on 31 December 1989, the intra-party hardliners were seen as a much bigger problem than the opposition (Jović 2003, p. 52).

This was the environment in which the ruling party embarked on the process of bringing republican electoral legislation in line with the 1988 changes to the federal constitution. The blueprint for the new legislation created by the expert group entrusted with drafting the new law by the Parliamentary Committee for the Socio-Political System left the non-democratic essence of the electoral system unchanged by keeping the nomination process intact. Nevertheless, these blueprints did offer some novelties. The lowest chamber of the Croatian Parliament (*Sabor*) was to be elected from an open party list. All candidates, however, would still be pre-validated in highly partisan local nomination processes designed to weed out the opposition.^7^ At the urging of local organisations of the socialist apparatus, the national list provision was abandoned and Croatia was divided into eight multimember districts based on its geographical regions with district magnitudes ranging from four to 20, with the average district magnitude being ten.^8^

One day before this new law was passed in the *Sabor* on 19 December, the Parliamentary Committee tried to legitimise it by inviting representatives of some of Croatia’s emerging (and still technically illegal) opposition groups for a quasi-roundtable discussion. The transcript of this meeting reveals the profound lack of understanding of the historical moment by many representatives of the regime. Members of the opposition warned them that their law was undemocratic and a sign the system was not truly for multiparty elections. No one discussed the open-list proportional representation elements implied in the electoral system because they were meaningless without a liberalised nomination process, and it was clear that even this deeply flawed law had its detractors among the hardliners in the Parliamentary Committee and in the government cabinet. Regime hardliners suggested that the popular sentiment was in favour of stability under their rule because ‘even the East Germans were now warning that their society was sliding into anarchy’. Other committee members, however, were fearful of the consequences of implementing an undemocratic electoral system. One was recorded in the minutes as saying, ‘I am really scared . … Do not, comrades, rigidly interpret [the constitution] . … Let these wretched [opposition] organisations and associations in’.^9^

In the end, the law was passed as drafted, though crucially without the accompanying regulation defining the multimember districts. This was put on hold because the attention turned to the 11th SKH Congress, which produced the SKH leadership that would face Milošević and his allies at the upcoming 14th Congress of the SKJ that January. In an attempt to solidify its base and garner support with the congressional representatives, the dogmatist wing of the SKH made calls for early elections in January 1990 under the barely revised electoral rules, something that the Hungarian ruling party had attempted to pull off earlier that spring but had not succeeded due to mass protests (Benoit & Schiemann 2001). The hardliners’ gambit, however, failed and the liberal wing under Ivica Račan narrowly won control of the party leadership. Rather than pushing for early elections, the Račan liberals opted to wait until the regular spring date, because they needed time to consolidate their grip on intra-party structures in preparation for the SKJ Congress.

On 11 January 1990—two weeks after the promulgation of the electoral law—the new liberal Central Committee of the SKH initiated the drafting of new electoral rules. It called for revisions of the constitution to accommodate the changing of the nomination rules and electoral procedures. The Parliamentary Committee for the Socio-Political System convened a new expert group the next day to set some guidelines. Although this expert group supposedly represented a variety of political views, its independence from the ruling party in formulating the electoral law was questionable (Kasapović 1997). Its head was Professor Smiljko Sokol, the dean of Zagreb University’s Faculty of Law, who was a member of the League of Communists. The transcript of their initial meeting of 12 January 1990 with the Parliamentary Committee reveals that they held a variety of views. The group had in front of it the law passed just days earlier, which instituted PR rules for elections to the lowest house, as well as the recently promulgated Slovenian electoral law, which also instituted a set of complex PR rules in multimember districts with national-level compensatory seats and voting panachage, allowing voters to choose candidates from different parties rather than from a party list (Krivic 1990). Proportional representation, therefore, seemed to be the norm of the time. It was also openly promoted by some members of the expert group as a system that could lead not only to the proportional representation of political groups but also ethnic minorities, a direct nod to Rokkan’s first path towards the institution of PR. However, the leaders of the Parliamentary Committee and Sokol, the head of the expert group, insisted that all electoral system options were on the table. As an expert on the French Fifth Republic, Sokol promoted French-style double-ballot rules, ostensibly to moderate electoral competition: ‘Wise people of the opposition should be persuaded not to play with fire that could not only burn down communism and take down a group of people from power but would also burn them’.^10^

Transcripts from the two subsequent meetings of the expert group with the Parliamentary Committee on 18 and 24 January reveal how exactly Sokol believed the French double ballot would help cool tempers and offer additional benefits to those in power. In his rather convincing argument, following Maurice Duverger, Sokol focused on the projected psychological rather than mechanical effects of the double ballot: French-style elections in two rounds promoted less extremist voting and campaigning because it was sensible for candidates to pursue the median voter and not antagonise potential partners prior to the second round. Elections under PR rules, according to Sokol, were run by national-level leaders who created party lists of loyalists running under banners of unified parties. Croatia did not need such national-level leaders, and his sponsors in the newly installed SKH leadership did not control a unified party anyway. They could not even produce a coherent national party list without leading to further breakdown of the local party branches.^11^ Implementing PR may have been a credible strategy for improving internal discipline by party leaders in some of Europe’s oldest democracies (Cox *et al*. 2018), but that strategy was not available to the liberal leadership of the SKH in the winter and spring of 1990.

Sokol was particularly taken by the Weimar example, suggesting that Hitler never could have come to power without PR. He was also likely affected by the French experiment with PR in the 1986 election that brought a significant increase in representation for Jean-Marie Le Pen’s *Front National*. The implication of his lengthy monologues in the meetings of the expert group and the Parliamentary Committee was that a PR system would benefit the opposition groups that were led by nationally recognisable personalities but did not have locally recognisable leaders, as was the case with Franjo Tuđman’s Croatian Democratic Union (*Hrvatska demokratska zajednica*—HDZ) and the Coalition of National Accord (*Koalicija narodnog sporazuma*—KNS), which featured a number of nationally known communist dissidents, but whose local candidates were basically unknown. A double-ballot system, on the other hand, would allow the ruling party to run an array of locally better-known personalities. This aspect of the system appealed particularly to the party bigwigs on the committee. One remarked, when asking the expert group to include provisions for MPs to be stripped of their seats if they chose to leave their party, ‘The proportional system highlights the role of the party. We have had that thus far and we have seen where it has brought us. A majoritarian system, on the other hand, highlights the quality of individual candidates and their programme’.^12^ The SKH label was no longer an asset. Equally importantly, these locally recognisable candidates of the ruling party could compete not only against the opposition but also among each other in the first election round. They would then ‘rationally’ opt for the more successful one among them before the second round. In other words, a two-ballot electoral system was to serve as a form of a primary: an unofficial electoral purge of the SKH ranks that were independent of the new party leadership but could not be dismissed so easily. As Sokol put it, in the first round both voters and candidates were expected to act with their hearts. In the second round, they were expected to act with their heads (Jakšić 1990).

The rest of the expert group and the Parliamentary Committee ultimately agreed with Sokol and the new law was passed, together with amendments to the republic’s constitution. Croatia got a system of two-round majority/plurality rules where candidates would qualify for the second round with 7% of the vote if no candidate earned a majority in the first round. They publicly defended the law as a simple mechanism for deciding parliamentary majority, but they also made it clear that the new president of the SKH’s Central Committee, Ivica Račan, played an ‘indispensable role’ in the formulation of electoral rules and the expediting of the process of electoral law design, which in the end took only four days.^13^

In some ways, the motivation of the new liberal SKH leadership behind pushing for this electoral system fit the East European pattern observed by other scholars: the SKH leadership believed they were better organised than the opposition; they anticipated that the local *nomenklatura* bosses would maintain their grip over rural voters; and in single-member districts, candidates would run on their personal name recognition. The SKH was indeed thought to have the best organisational structure, although that was highly doubtful, especially in light of remarkable organisational feats of the HDZ in the months leading up to the elections that were plain for all to see in this party’s mass rallies and well-attended membership drives (Kasapović 2014). The communists also included a number of eminent public personalities on their slates in order to lower the liabilities of a communist label. The crucial factor, however, was the ruling party’s internal division and the weakness of its leadership in disciplining local branches, coupled with the fact that the opposition parties were themselves divided between two blocs: the HDZ and the KNS.

The two-ballot system was confirmed as the electoral system of choice just two days after Ivica Račan pulled the majority of Croatia’s delegates from the SKJ’s 14th Congress on 22 January, with a substantial number of Croatian Serb delegates remaining behind, leading the Belgrade press to suggest these local functionaries would abandon the SKH and form their own party (Šentija 2005, p. 100). The leading Yugoslav weekly, *Danas*, captured the situation in the SKH with an article titled ‘The Schism of Croatian Communists: How to Win Elections and Not Lose the Serbs’ (Marinković 1990). The chosen electoral system allowed Račan not to lose the Serbs or the conservative wing of the party by allowing them to run in electoral districts alongside the more liberal candidates loyal to his leadership, all while leaving him with the prospects of winning enough seats in the second round against the opposition that was itself divided into two blocs.

Ultimately, the implemented rules proved to be a disaster for the communists from the perspective of seat maximisation. The opposition may have been divided between the HDZ and the KNS, but the dichotomous nature of the campaign favoured the strongly anti-communist and anti-Milošević platform of the HDZ. The communists presented a divided front, not only of three parties—the SKH (which added to its name the suffix of ‘Party of Democratic Change’, *Stranka demokratskih promjena*—SDP), the Socialist Alliance (*Socijalistički savez–Savez socijalista Hrvatske*—SS–SSH) and the League of Socialist Youth (*Savez socijalističke omladine Hrvatske*—SSOH)—but also of a multitude of candidates running in the same electoral districts. Out of 80 districts for the lowest house of the *Sabor*, system parties ran single candidates in only 27. In 12 districts, there were even multiple SKH candidates: those loyal and disloyal to the new party leadership.

On the eve of the election, the communist candidate running in Franjo Tuđman’s district, Marija Šola, stated that she ‘hoped that the heart does not overrule the mind’ (Šola 1990). It was a clear reference to Sokol’s defence of the electoral rules he helped design. Šola’s worries were well-founded, since the electoral rules led to massive overrepresentation of the HDZ in the elections held on 22 April and 6 May. With 42% of the vote, they won 69% of the seats. The communists and their allies, on the other hand, won 34% of the vote (26% for SKH and 8% for its satellites) and only 24% of the seats. Ivica Račan recognised between the two rounds that his choice in the trade-off between seat maximisation and maintaining intra-party unity was a mistake, but it was too late (Babić 1990). Neither did the electoral system prevent the eventual dissolution of the SKH, which in many ethnically mixed localities fell apart after the elections. A substantial number of its local members and leaders later joined the Serb rebellion or—on the Croatian side—joined other parties (Filipović 2019), among them, Smiljko Sokol. After a barrage of criticism from his party colleagues, he left the SKH for the HDZ and became one of the main architects of Croatia’s first democratic constitution and a government minister (Ribičič 1995, p. 28).

Although the electoral system chosen by the newly installed liberal leaders of the Croatian communists is commonly and rightfully seen as a mistake from the perspective of seat maximisation, the logic behind their choice was clear. They needed to find the balance between keeping intra-party peace and prospects for electoral victory. The electoral rules offered them a chance to weed out internal opposition without direct antagonism in the first round while facing external opposition divided between the HDZ and the KNS in the second round. Proportional representation may have been considered the norm at the time, in light of the Slovenian communists’ choice of electoral system and the electoral rules already in place in Croatia. By ‘reading history forward’ and unpacking the SKH into its constituent parts rather than considering the ruling party a unitary actor, we can understand why the Croatian communist leadership opted for something completely different from PR. Internal divisions and the nature of the opposition led it to adopt the French-style two-round system. Their electoral failure does not detract from the rationality of that choice.

## Bosnia–Hercegovina: cushioning the fall with proportional representation

The platform for the recentralisation of Yugoslavia by the Milošević regime in Belgrade resulted in deep cleavages within the Bosnian League of Communists (*Savez komunista Bosne i Hercegovine*—SKBiH), which were—as in Croatia—more often than not ethnically based. The SKBiH membership was disproportionally Serb, whereas its leadership was balanced between the republic’s three ethnic groups: Muslims, Serbs and Croats (Andjelic 2003, p. 130). Moreover, the leadership of SKBiH was destabilised after a string of financial scandals took down a whole generation of power-holders in the late 1980s. The newly installed leaders, including the president of the SKBiH Central Committee, Nijaz Duraković, tried to secure legitimacy of their rule by being ideologically purist (that is, committed to both socialism and Yugoslavism), while maintaining a balance between the two dominant camps—Serbian and Slovenian—in the federation (Lovrić 1989b).

Throughout 1989, the ruling party was engaged in the process of bringing electoral legislation in line with the 1988 changes to the federal constitution, as were the other republics. This process led to a new electoral law on 28 December 1989 that was similar, though not identical, to the one simultaneously implemented in Croatia (Skupština SRBiH 1989a). The crucial difference was that the Bosnian communists prescribed the exact number of candidates that had to be nominated by the different organisations of the system—the League of Communists, Socialist Alliance, Confederation of Labour Unions (*Savez sindikata*), Alliance of Organisations of National Liberation War Fighters (*Savez udruženja boraca Narodnooslobodilačkog rata*—SUBNOR) and the League of Socialist Youth—and agreed to by all of them within the nominating process to be organised by the Socialist Alliance (Article 58). As in Croatia, the lowest Socio-Political Chamber was to be elected from an open national list, and the SKBiH kept its grip on power by controlling the nomination process (Skupština SRBiH 1989b). Elections were projected to take place in March 1990, that is, two months after the 14th Congress of the SKJ.

Three events, however, proved that the communists’ hope of retaining power was futile. First, the reactions to the new electoral law within the various organisations of the system, as well as the leading media houses that were growing independent of the communist leadership, were negative (Grković 1989; Habuz & Stanišić 1989). They suggested that the law did not reflect the democratising reality in Bosnia–Hercegovina, Yugoslavia or the rest of Eastern Europe. Second, the 10th Congress of SKBiH, held in advance of the SKJ congress, demonstrated the depth of the rift between the liberal/federalist and dogmatist/centralist camps. Though their differences were papered over in a bland declaration, the congress showed that the SKBiH was anything but a unified organisation and that its leadership had little grip on power within the party. And finally, the schism between the Slovenian and Croatian communists on the one side and the Serbian communists and their allies on the other at the 14th Congress of the SKJ made the position of equidistance played by the SKBiH leadership untenable.

At the 14th SKJ Congress and in the months that followed, it seemed that the SKBiH leadership under Duraković would fall in line behind Milošević. During his speech at the Belgrade congress, Duraković showed the door to all party ‘heretics’ who were suggesting the SKJ was finished. Though he later tried to suggest otherwise, his theatrics were correctly interpreted as directed against both the Slovenes and the reformers within the SKBiH. Duraković subsequently doubled down on placating the dogmatist wing of his party. On 21 February 1990, the Assembly of Bosnia–Hercegovina passed a new Law on Citizens’ Associations banning all parties organised on an ethnic basis. The SKBiH also postponed the planned March elections and announced that it would participate in the resurrection of the 14th SKJ Congress under Milošević’s leadership later that May. On 25 May—the date that used to be celebrated as the birthday of Tito—it held a mass rally of supporters on the streets of Sarajevo. Duraković addressed the crowd by asserting that Bosnia’s new democracy would have limitations: ‘We are for democracy … but there will be no democracy for nationalists and neofascists’ (Živković & Habul 1990). The rally also served as a mobilising effort in advance of Duraković’s SKBiH participating in the rump SKJ congress together with Milošević and his acolytes in Belgrade the following day.^14^

The strategy of embracing the hardliners, however, proved unsuccessful. Internal documents of the republic’s electoral commission under SKBiH control showed that running semi-free elections would be virtually impossible in a number of localities because there was no interest. Out of 109 Bosnian municipalities, 22 did not have any registered electoral candidates for republican positions, and 49 did not have any candidates for federal positions. This was, according to the commission, a worrying indication that political engagement from the party base was in many regions almost non-existent.^15^ Unlike the liberal leadership of the Croatian communists, which had to field multiple candidates in individual districts to maintain a semblance of internal unity by placating the conservative wing of the party, Bosnian communist leadership faced a completely different problem: it could not recruit candidates to run in about one third of the republic because its dogmatism turned off significant parts of the party base and the electorate. In the immediate aftermath of the 14th SKJ Congress, it was already clear that the liberal wing of the party would not forgive Duraković his performance (Kamenica 1990). In the months that followed, many of these reformist communists either became politically inactive or joined the party’s new challenger from the liberal left: the Alliance of Reformist Forces of Yugoslavia (*Savez reformskih snaga Jugoslavije*—SRSJ) led by the federal prime minister, Ante Marković, himself of Bosnian Croat descent (Filipović 2021).

The importance of the formation of the SRSJ in the late spring and early summer of 1990 for the nature of the electoral institutions ultimately designed by the SKBiH and the character of the electoral campaign that would unfold that fall cannot be overstated. The SRSJ poached a number of competent reformist SKBiH functionaries. It also changed the dynamics of the electoral landscape by presenting a credible challenge to the ruling party from the left. Marković and his federal programme of economic stabilisation and reforms were popular in Bosnia–Hercegovina. By embracing the dogmatist wing of the League of Communists, Duraković essentially split the party and provoked Marković’s entry into the campaign, with disastrous effects on SKBiH’s electoral prospects.

Duraković’s strategy collapsed in the summer of 1990. On 12 June, the Constitutional Court struck down the February Law on Citizens’ Associations, making it clear that the communist leadership no longer had control over the judiciary.^16^ Ethnically based political parties—Party of Democratic Action (*Stranka demokratske akcije*—SDA) for the Muslims, Serb Democratic Party (*Srpska demokratska stranka*—SDS) for the Serbs and Croatian Democratic Union (*Hrvatska demokratska zajednica*—HDZ) for the Croats—began to form and mobilise in plain sight of the authorities in spite of the ban, and there was little that the communists could do to stop it. With Ante Marković holding a rally of 100,000 people to officially launch his party on 29 July, the only thing that the communists could do was to acknowledge reality and minimise their losses by passing a new set of electoral laws two days later on 31 July (Skupština SRBiH 1990).

Although the Assembly was ostensibly still filled with members of only one political option, the debate on these new electoral laws was discordant. After a month-long public consultation, which showed the local organisations of the socialist system divided on the question of new parliamentary institutions,^17^ the SKBiH leadership came out with a proposal for the new lower house, named the Chamber of Citizens, to be elected under a system of proportional representation in seven relatively large multimember districts matching the republic’s regional chambers of commerce. There was no doubt why the SKBiH leadership wanted proportional representation. In his press interviews, Nijaz Duraković made it clear that he learned from the choices made by his Croatian counterparts (Mikulandra 1990). The official documents prepared for the parliamentary debate by the narrow circle of party officials who drafted the new electoral law (unlike in Croatia, there was no expert group) also stated that the law was created based on observations made in Slovenia and Croatia.^18^ Although public opinion polls in Yugoslav media were inaccurate and biased, they were already showing a highly fractured Bosnian electorate where Duraković’s communists could at best hope for a third of the votes, with both the reformists and the ethnic parties quickly catching up (Malešević 1991). A testament of the level of confidence regarding electoral results within the SKBiH leadership was the question of electoral threshold. The law’s architects suggested a 2% threshold in their final draft, but even this mild measure of disproportionality was dropped from the law that was ultimately adopted.^19^ This was done to minimise SKBiH’s projected losses by maximising proportionality and dispersion of seats in the new parliament, as well as by making entry into parliament easier for the communists’ minor satellites in the Socialist Alliance (now relabelled the Democratic Alliance of Socialists (*Demokratski savez socijalista*—DSS)) and the Alliance of Socialist Youth (*Savez socijalističke omladine*—SSO), which decided to contest the elections separately from SKBiH. Unlike the Croatian communist leadership, which used the first round as a form of primary election, fielding disloyal candidates in single-member districts to effectively eliminate them from seats, the SKBiH leadership under Duraković could turn to proportional representation because those who did not approve of its platform already left the party for the SRSJ (or, in some cases, for the ethnic parties).

The electoral campaign and the results of the elections held on 18 November proved that the SKBiH leadership had chosen the correct electoral system. Ethnically based parties thrashed the communists and reformists, which were too busy fighting each other for the same sliver of the electorate. Although there were attempts to cut some sort of a deal between the SKBiH and SRSJ during the campaign and to create a coordinated response to the nationalists, this came to nothing, primarily due to animosity between the leaders of the two parties stemming from the days when they were all highly placed communist functionaries (Andjelic 2003, p. 180). The ethnically based parties, on the other hand, were united in their common goal of overthrowing communists. Due to the ethnic makeup of Bosnian municipalities, which most often favoured one of the three dominant ethnic groups, the SDA, SDS and the HDZ were able to carve up different regions of the republic with relative ease, leading to a catastrophic result for the left. In the lower house, communists and their allies received 15% of the votes (12% for the SKBiH and 3% for its satellites) and 13% of the seats (the abolition of the 2% threshold helped both the DSS and the SSO get one and two representatives respectively). The reformists fared even worse. In the lower house, they received 9% of the votes and seats. The ethnic parties—SDA, SDS and HDZ—swept to power and formed a coalition government. The Bosnian communists’ result was comparable to that of their Hungarian and Czechoslovak counterparts. Unlike the Hungarian and Czechoslovak communists, however, Bosnian communists lost to a coalition of nationalist parties, which set the republic on course toward ethnic divisions and, ultimately, war.

The decision of the SKBiH leadership to push for proportional representation in the lower house elections was obviously a reflection of the calculus based on the local balance of forces and on the lessons learned from Slovenia (where the communists were in a similar strategic situation and opted for PR of high proportionality) and Croatia in particular. Unlike the liberal leadership of the Croatian communists, which faced an oversupply of (both loyal and disloyal) candidates, the leadership of SKBiH under Nijaz Duraković was faced with an exodus of a whole generation of liberal party functionaries who opted for the SRSJ because they did not approve of Duraković’s courting of the hardliners and the Serbian leadership under Slobodan Milošević. Moreover, unlike the Croatian communists who faced what seemed to be a divided opposition led by the HDZ and the KNS, the SKBiH additionally faced a *de facto* united right-wing opposition of the three main ethnic parties. Proportional representation in the lower house elections cushioned their collapse, which was, nevertheless, the most dramatic of all communist downfalls in former Yugoslavia.

## Serbia: reaping the benefits of majoritarian disproportionality

Unlike the Leagues of Communists of Croatia and Bosnia–Hercegovina, the League of Communists of Serbia was anything but riven by divisions in 1989 and 1990. Under the leadership of Slobodan Milošević, who became president of the Presidency of its Central Committee in 1986, the League of Communists of Serbia was purged of all dissident voices in 1987 and 1988. Milošević and his associates created a platform for the solution of Yugoslavia’s economic and political crisis that was highly popular in Serbia. This platform could best be labelled recentralisation: first of Serbia, by limiting the autonomies of Vojvodina and Kosovo, and then of the Yugoslav federation. Faced with an intensifying campaign of demonstrations by Kosovo Serbs, who were protesting alleged discrimination by Kosovo’s Albanian majority in 1986 and 1987, the Milošević regime coopted their nationalist platform and used it to re-legitimise its rule, as well as to eliminate opponents of its leadership within the party.

Throughout 1988 and 1989, the demonstrations by Kosovo Serbs were dramatically amplified by the ruling party, turning into the largest protest campaign in Eastern Europe, bringing millions of people to the streets (Ramet 2006). This campaign, which became known as the ‘anti-bureaucratic revolution’ (Grdešić 2019), presented not only a vision of a reformed and recentralised Serbia and Yugoslavia, but also one of a new structure of political representation in which the ruling party would openly embrace nationalism and continue its domination in a system of supposedly ‘non-partisan pluralism’. With the help of the Serbian media houses and intellectual elites (Dragović-Soso 2002), Milošević established unparalleled control over all levers of political power within his party and republic. His regime also successfully sabotaged the development of the nascent opposition groups, which remained weak and divided for the better part of the decade to come (Ramet 2006).

Just like Croatia and Bosnia–Hercegovina, Serbia passed a new electoral law in the run-up to the 14th Congress of the Yugoslav League of Communists. Unlike Croatia and Bosnia–Hercegovina, however, Milošević’s Serbia actually held elections under these, still undemocratic, rules in November of 1989. The new rules remained essentially the same as in the ‘delegate system’, except that voters could now also directly elect their representatives to the parliament of the republic—the National Assembly—and they could express their views on the candidates for the post of the president of the republic’s presidency in a concurrent consultative referendum. The lowest house of the parliament was elected in a version of partially open-list PR elections where the whole republic was one electoral district, but different institutions of the system (such as the League of Communists, Socialist Alliance, labour unions and youth organisations) put forward parts of the common national list. The electoral process was still closed to the opposition through the usual limitations in the nominating procedures (Romanić 1989). Unsurprisingly, Milošević won the consultative referendum vote for the president of the republic’s presidency, with more than 80% of the vote and turnout curiously exceeding 100% in some municipalities.

The collapse of the federal League of Communists that January and the string of democratic elections that swept over Eastern Europe—including other Yugoslav republics—throughout 1990, made the prospects of the Serbian National Assembly serving out its full term untenable. Croatian and Bosnian communists passed the minimum constitutional changes needed to hold democratic elections and left the full constitutional redesign for the first democratically elected parliaments. Serbian communists under Milošević chose a different path. They wanted to lock in what they saw as their principal achievement in the form of constitutional restrictions on the autonomous status of Kosovo and Vojvodina prior to elections and decided to bind the passing of electoral reforms to the promulgation of the new constitution. It was a trap for the emerging opposition parties because their disapproval of the electoral system proposed by the communists could be portrayed by the ruling regime as disapproval of the constitutional ‘reunification’ of Serbia. Milošević even put the issue of passing of the new constitution prior to the democratic elections to the voters in a referendum held on 1–2 July 1990. Voters overwhelmingly supported his position with 97% in favour, with a 76% turnout. The clear result of the referendum encouraged the regime to push through its preferred set of electoral rules: its true bone of contention with the opposition.

The opposition parties were clearly cognisant of their standing in the electorate and the uphill battle they had to fight against the Milošević juggernaut. This is why they were united in their proposals for a PR system with the highest possible dose of proportionality. Their proposals, whether in the form of one national list or several relatively large regional districts, were ironically very similar to the system used for the 1989 elections to the lowest house of the Serbian National Assembly, with three crucial differences: nomination procedures were to be liberalised and opened to parties and lists of independent candidates; the number of MPs was to rise from 90 to more than 200 in order to further increase parliamentary access for smaller parties; and seats were to be allocated to lists based on their results rather than to individual candidates of communist-system organisations (Jovanović 1997, pp. 129–30).

The communists, on the other hand, wanted a complete departure from proportional representation and opted for a two-round majority runoff system whereby Serbia was to be divided into 250 single-member districts. Their proposed legislation also included a number of provisions that could, at best, be labelled electoral gamesmanship by the ruling regime. For example, candidates needed to collect 500 signatures—that is, on average 2% of registered voters in a district. There was also no guarantee of opposition parties’ participation in the monitoring of local electoral commissions or of their equal access to public media (Jovanović 1997, p. 131). The opposition parties announced a possible boycott and demanded roundtable negotiations with the communists. Although the ruling party made some changes to the legislation, the call for roundtable negotiations was rebuffed and the majoritarian rules remained.^20^ Moreover, the electoral districts were heavily gerrymandered with dramatic variation in size from 6,240 to 46,642 voters.^21^

The reasons for the regime’s choice of majoritarian electoral rules were simple. Over the previous several years of the ‘anti-bureaucratic revolution’, Milošević had secured full control over the ruling party and system. That summer, he formalised it by merging the League of Communists of Serbia with the Socialist Alliance into the Socialist Party of Serbia (*Socijalistička partija Srbije*—SPS). By doing that, he inherited not only the physical resources of the Socialist Alliance but also its administrative reach into all corners of Serbia’s social and political life. He also ensured full control over the whole left end of the political spectrum. The opposition, on the other hand, was weak, divided and simply outplayed by the regime. A clear glimpse into the mindset of Milošević and his associates is provided by the diary of Borisav Jović, Serbia’s representative in Yugoslavia’s federal presidency at the time and one of Milošević’s closest collaborators. In a string of meetings between Milošević and his inner circle throughout 1990, Jović noted that the Serbian leader expressed no doubts that he and his party were going to win the elections and that the opposition presented no real challenge to his rule. On the eve of elections, Milošević apparently believed his party would win 60% or more seats in the National Assembly.^22^

He was proved correct. Milošević’s party ran a masterful campaign under the slogan of ‘With us, there is no uncertainty’, which appealed to wide swathes of the electorate yearning for stability. The SPS trounced the opposition divided between the Serb Renewal Movement (*Srpski pokret obnove*—SPO) of Vuk Drašković, the Democratic Party, and various other independent and minority groups. The two-round majoritarian rules brought a massive boost to the ruling party, which won 78% of the seats on 46% of first-round votes in the elections held on 9 December, with the second round held on 23 December. The contrast between Milošević’s SPS and the communists in Croatia and Bosnia–Hercegovina could not have been starker. Milošević built a disciplined electoral machine, running single candidates loyal to his leadership in each electoral district. The SPS particularly benefited from the electoral boycott by Kosovo Albanians, winning 30 out of 34 seats from this province. Its choice of the majoritarian electoral system, rooted in its internal cohesion and strength in contrast to the weakness and division of the nascent opposition, proved to be not only a rational but also an effective decision.

## Conclusions

When Arend Lijphart declared, back in 1992, that Rokkan’s explanations of the adoption of PR in Western Europe during the first wave of democratisation were the best explanations for the constitutional choices in Eastern Europe during the third wave of democratisation, he was onto something (Lijphart 1992, p. 207). In this article, we have argued that the logic behind the institutional preferences of the communist elites was definitely Rokkanian. After all, it was the communist power-holders who had the most to lose. They not only faced potential electoral defeats but social irrelevance or worse through possible lustration or retribution. Our in-depth analysis of the communist leaderships in the three largest Yugoslav republics clearly confirms that the main driver of their electoral system preferences was the balance between their intra-party unity/strength and the electoral threat by the opposition. In Serbia, the communist power-holders had firm intra-party control and simultaneously faced a weak opposition, so they opted for majoritarian rules. In Bosnia–Hercegovina, the communist leaders had a weak grip over a divided front of system parties, and they faced an organised leftist challenge from the Alliance of Reformist Forces and a strong and coordinated opposition, so they opted for PR rules of high proportionality. In Croatia, the balance of power was uncertain, so the communist leadership opted for electoral rules that were supposed to provide them with strategic intra-party and electoral benefits.

Extending this argument to the remaining three republics is relatively straightforward. The parallel between the strategic position of Slovenia’s communist leadership and the position of the leadership of Bosnia–Hercegovina is clear. Slovenian communists opted for a PR system of high proportionality because they were also divided (in their case, into three distinct parties growing out of the organisations of the socialist system) and had to face a strong opposition united in the Demos coalition. The Macedonian communist leadership, on the other hand, opted for the same French-style majority-plurality electoral system as the Croatian communist leadership because they did not have full control of the system candidates either and wanted to use first-round elections as primaries, while at the same time facing a divided opposition of unclear strength. Indeed, system parties in Macedonia did not run multiple candidates in only seven out of 120 districts and the nationalist Internal Macedonian Revolutionary Organisation–Democratic Party for Macedonian National Unity (*Vnatrešna makedonska revolucionerna organizacija–Demokratska partija za makedonsko nacionalno edinstvo—*VMRO–DPMNE), which ultimately emerged with the plurality of seats, only recovered in the second round when it received 30% of the votes, as opposed to just 14% in the first round. The only curiosity in this context may appear to be the decision of the Montenegrin communist leaders, considering the strength of their party, to opt for a PR system with a wide range of district magnitudes (from 1 to 29 seats), but we believe one of the crucial factors here was the robust leftist challenge by the SRSJ and the strategic benefits districts of such widely differing sizes may bring. More in-depth archival work is needed to fully explain this case. The take-home message from our study, however, is clear: if we do not focus on ruling parties as unitary actors but on their leadership instead, and if we understand their power goals more broadly than seat maximisation, then Rokkan’s strategic calculus used to explain electoral system formation during the first wave of democratisation indeed does offer a solid framework for understanding institutional choices of the ruling elites during the third wave of democratisation.

In addition to demonstrating the value of a classical theoretical approach, while identifying refinements needed to bring theory closer in line with political practice, we believe our article also makes two other important contributions to the literature. First, it highlights the value of the Yugoslav republics as cases in the study of institutional formation during early transition from communism. Yugoslav republics are, unfortunately, often completely neglected by the comparative literature, likely due to their eventual collapse into violent conflict, even though they lend themselves very nicely to comparative study, particularly given their uniformity in institutional structures prior to the end of communism and vastly different institutional choices at the time of transition into multipartyism. Second, we believe the value of our study also lies in its historicisation of a crucial period in the development of political institutions in Eastern Europe. With the transitions from communism passing the 30-year mark most often needed for the opening of the archives, the time is coming to properly test many of our initial conceptions of the transition from communism by using primary sources and ‘reading history forward’. We hope our study gives other scholars the impetus to revisit this crucial period in the history of East European political development.

## Footnotes

1 The Yugoslav republics barely rated a mention in influential monographs and edited volumes on institutional design in the early transition (Lijphart & Waisman 1996; Elster *et al*. 1998; Birch *et al*. 2002) or in the general literature on elections and voting during this time (Tucker 2002), and there has been little improvement since.

2 While cognisant of these idiosyncrasies, for the sake of brevity, we refer to Yugoslav ruling party/parties and their elites as communists. After all, that is what they called themselves.

3 We conducted archival research in the Archive of the Croatian *Sabor* (*Pismohrana Hrvatskog Sabora*) and the Archive of Bosnia–Hercegovina (*Arhiv Bosne i Hercegovine*) where we reviewed more than 2,000 pages of relevant archival materials. In both archives we were forced to rely on the goodwill and memory of archival staff because the collections were, in most cases, either uncatalogued or poorly organised. Materials in the Archive of Bosnia–Hercegovina were in a particularly disappointing state of disarray after the 2014 fire, making this kind of research extremely difficult and time-consuming.

4 For a notable counterargument suggesting West European electoral system designers operated under similar conditions of uncertainty, see Andrews and Jackman (2005). For an important exception to the argument that East European parties were unitary actors, see Birch *et al*. (2002). However, they do not offer a systematic explanation for electoral system design but instead highlight country-specificity and strategic diversity among cases (Birch *et al*. 2002, p. 20).

5 District magnitude is ‘the number of legislative seats assigned to each electoral district' (Rae 1995, p. 65).

6 ‘Top lista hrvatskih političara’, *Danas*, 17 October 1989.

7 Archive of the Croatian *Sabor*, ‘Nacrt Zakona o delegatskim izborima’, Republička konferencija SSRNH and Vijeće Saveza sindikata Hrvatske, March 1989.

8 ‘Prijedlog za donošenje zakona o određivanju izbornih područja za izbor zastupnika u Društveno-političko vijeće Sabora Socijalističke Republike Hrvatske’, *Delegatski vjesnik*, 9 December 1989.

9 Archive of the Croatian *Sabor*, ‘Zapisnik sa 47. sjednice Odbora za društveno-politički sistem Društveno-političkog vijeća Sabora SR Hrvatske’, 18 December 1989, 20/6/VM–20/8/VM.

10 Archive of the Croatian *Sabor*, ‘Zapisnik sa sastanka Odbora za društveno-politički system—Radna grupa za pripremu promjena Zakona o izboru i opozivu odbornika i zastupnika’, 12 January 1990, 2/10/JP.

11 Archive of the Croatian *Sabor*, ‘Zapisnik sa sastanka Radne grupe Odbora za društveno-politički sistem Društveno-političkog vijeća Sabora Socijalističke Republike Hrvatske’, 18 January 1990, 7/6/LJ–8/4/HLJ.

12 Archive of the Croatian *Sabor*, ‘Zapisnik sa sjednice Odbora za društveno-politički sistem Društveno-političkog vijeća Sabora Socijalističke Republike Hrvatske’, 24 January 1990, 3/1/JG.

13 ‘Prvi demokratski izbori u povijesti Sabora’, *Vjesnik*, 27 January 1990.

14 ‘Računamo na demokratski rasplet’, *Oslobođenje*, 27 May 1990.

15 Archive of Bosnia–Hercegovina (uncatalogued), ‘Informacija o sprovedenim izbornim aktivnostima za izbor delegata u skupštine društveno-političkih zajednica i o utrošenim sredstvima—mart 1990. Godine’, Republička izborna komisija SRBiH, April 1990.

16 Archive of Bosnia–Hercegovina (uncatalogued), ‘Odluka Ustavnog suda SRBiH za ocjenjivanje ustavnosti odredaba čl. 4 stav 2 i čl. 8 Zakona o udruživanju građana’, Ustavni sud SRBiH, 12 June 1990.

17 Archive of Bosnia–Hercegovina (uncatalogued), ‘Izvještaj o rezultatima javne diskusije o nacrtu amandmana na Ustav SR BiH’, Skupština SRBiH,10 July 1990.

18 Archive of Bosnia–Hercegovina (uncatalogued), ‘Predlog alternativnih rješenja za Predlog Zakona o izboru odbornika i poslanika u skupštine drušveno-političkih zajednica u slučaju da se amandmanima na Ustav SRBiH utvrdi dvodomna struktura Skupštine SR BiH’, Izvršno vijeće Skupštine SRBiH, July 1990.

19 Archive of Bosnia–Hercegovina (uncatalogued), ‘Predlog Zakona o izboru odbornika i poslanika u skupštine drušveno-političkih zajednica’, Izvršno vijeće Skupštine SRBiH, July 1990.

20 ‘U Srbiji počinje izborna trka’, *Politika*, 30 September 1990.

21 *Izbori 1990. Konačni rezultati izbora za predsednika republike i narodne poslanike* (Belgrade, Republički zavod za statistiku Srbije).

22 Particularly instructive are their meetings held on 10 January, 19 January, 21 March, 28 April, 7 June and 26 October (Jović 1996, pp. 88, 92, 124, 144, 152, 218). Jović’s diary was also used as evidence in the trial against Milošević at the International Criminal Tribunal for the Former Yugoslavia (ICTY).

## References

[CIT0001] Ahmed, A. (2010) ‘Reading History Forward: The Origins of Electoral Systems in European Democracies’, *Comparative Political Studies*, 43, 8/9.

[CIT0002] Andjelic, N. (2003) *Bosnia–Herzegovina: The End of a Legacy* (London, Frank Cass).

[CIT0003] Andrews, J. T. & Jackman, R. W. (2005) ‘Strategic Fools: Electoral Rule Choice Under Extreme Uncertainty’, *Electoral Studies*, 24, 1.

[CIT0004] Babić, J. (1989) ‘Što poslije peticija’, *Danas*, 19 December.

[CIT0005] Babić, J. (1990) ‘Iskoristit ćemo sve šanse’, *Danas*, 1 May.

[CIT0006] Balkovec, B. (2017) *Svi na noge, svi van, da pobjeda bude što sjajnija! Izborna teorija i praksa u međuratnoj Jugoslaviji* (Zagreb, Srednja Europa).

[CIT0007] Barzachka, N. S. (2014) ‘When Winning Seats is not Everything: Tactical Seat-Loss During Democratization’, *Comparative Politics*, 46, 2.

[CIT0008] Benoit, K. (2007) ‘Electoral Laws as Political Consequences: Explaining the Origins and Change of Electoral Institutions’, *Annual Review of Political Science*, 10.

[CIT0009] Benoit, K. & Hayden, J. (2004) ‘Institutional Change and Persistence: The Evolution of Poland’s Electoral System, 1989–2001’, *Journal of Politics*, 66, 2.

[CIT0010] Benoit, K. & Schiemann, J. W. (2001) ‘Institutional Choice in New Democracies: Bargaining Over Hungary’s 1989 Electoral Law’, *Journal of Theoretical Politics*, 13, 2.

[CIT0011] Birch, S., Millard, F., Popescu, M. & Williams, K. (2002) *Embodying Democracy: Electoral System Design in Post-Communist Europe* (New York, NY, Palgrave Macmillan).

[CIT0012] Boix, C. (1999) ‘Setting the Rules of the Game: The Choice of Electoral Systems in Advanced Democracies’, *American Political Science Review*, 93, 3.

[CIT0013] Boix, C. (2010) ‘Electoral Markets, Party Strategies, and Proportional Representation’, *American Political Science Review*, 104, 2.

[CIT0014] Braunias, K. (1932) *Das parlamentarische Wahlrecht: Ein Handbuch über die Bildung der gesetzgebenden Körperschaften in Europa. Vol. 2* (Berlin, W. de Gruyter).

[CIT0015] Cox, G. W., Fiva, J. H. & Smith, D. M. (2018) ‘Parties, Legislators, and the Origins of Proportional Representation’, *Comparative Political Studies*, 52, 1.

[CIT0016] Cusack, T. R., Iversen, T. & Soskice, D. (2007) ‘Economic Interests and the Origins of Electoral Systems’, *American Political Science Review*, 101, 3.

[CIT0017] Dragović-Soso, J. (2002) *Saviours of the Nation’: Serbia’s Intellectual Opposition and the Revival of Nationalism* (London, Hurst & Company).

[CIT0018] Elster, J., Offe, C. & Preuss, U. K. (1998) *Institutional Design in Post-Communist Societies: Rebuilding the Ship at Sea* (Cambridge, Cambridge University Press).

[CIT0019] Filipović, V. (2019) ‘Stranačka politika u Vukovaru, 1990–1991’, *Anali Hrvatskog politološkog društva*, 16, 1.

[CIT0020] Filipović, V. (2021) ‘“Markovićeva stranka”: Savez reformskih snaga Jugoslavije (Osnivanje, program i izbori 1990)’, *Politička misao*, 58, 1.

[CIT0021] Geddes, B. (1996) ‘Initiation of New Democratic Institutions in Eastern Europe and Latin America’, in Lijphart, A. & Waisman, C. H. (eds).

[CIT0022] Grdešić, M. (2019) *The Shape of Populism: Serbia Before the Dissolution of Yugoslavia* (Ann Arbor, MI, University of Michigan Press).

[CIT0023] Grdešić, I., Jantol, T., Kasapović, M., Šiber, I. & Tomac, Z. (1986) *Delegatski sistem 1974–1984* (Zagreb, Informator).

[CIT0024] Grković, L. (1989) ‘Pobrkan red poteza’, *Oslobođenje*, 15 December.

[CIT0025] Habuz, E. & Stanišić, D. (1989) ‘Odbijen zakon o izborima’, *Oslobođenje*, 21 December.

[CIT0026] Jakšić, M. (1990) ‘Većinskim sistemom do stabilnijeg parlamenta’, *Vjesnik—Panorama subotom*, 10 February.

[CIT0027] Jovanović, M. (1997) *Izborni sistemi: izbori u Srbiji, 1990–1996* (Belgrade, Službeni glasnik).

[CIT0028] Jović, B. (1996) *Poslednji dani SFRJ: Izvodi iz dnevnika* (Belgrade, Politika).

[CIT0029] Jović, D. (1989) ‘Zeleni ispred crvenih’, *Danas*, 26 December.

[CIT0030] Jović, D. (2003) *Jugoslavija, država koja je odumrla: Uspon, kriza i pad Kardeljeve Jugoslavije, 1974–1990* (Zagreb, Prometej).

[CIT0031] Kamenica, E. (1990) ‘“Mladobosance” ima tko da brani?!’, *Komunist*, 2 February.

[CIT0032] Kaminski, M. M. (2002) ‘Do Parties Benefit from Electoral Manipulation? Electoral Laws and Heresthetics in Poland, 1989–1993’, *Journal of Theoretical Politics*, 14, 3.

[CIT0033] Kasapović, M. (1997) ‘Parliamentary Elections in Croatia: Electoral Models and Their Effects’, in Šiber, I. (ed.) *The 1990 and 1992/3 Elections in Croatia: Analyses, Documents and Data* (Berlin, Edition Sigma).

[CIT0034] Kasapović, M. (2014) *Kombinirani izborni sustavi u Europi, 1945–2014* (Zagreb, Plejada).

[CIT0035] Kreuzer, M. (2010) ‘Historical Knowledge and Quantitative Analysis: The Case of the Origins of Proportional Representation’, *American Political Science Review*, 104, 2.

[CIT0036] Krivic, M. (1990) ‘Izbori u Sloveniji 1990’, *Politička misao*, 27, 2.

[CIT0037] Leemann, L. & Mares, I. (2014) ‘The Adoption of Proportional Representation’, *Journal of Politics*, 76, 2.

[CIT0038] Lijphart, A. (1992) ‘Democratization and Constitutional Choices in Czecho-Slovakia, Hungary and Poland 1989–91’, *Journal of Theoretical Politics*, 4, 2.

[CIT0039] Lijphart, A. & Waisman, C. H. (eds) (1996) *Institutional Design in New Democracies: Eastern Europe and Latin America* (Boulder, CO, Westview).

[CIT0040] Linz, J. J. & Stepan, A. (1992) ‘Political Identities and Electoral Sequences: Spain, the Soviet Union, and Yugoslavia’, *Daedalus*, 121, 2.

[CIT0041] Lovrić, J. (1989a) ‘Kome smeta Hrvatska’, *Danas*, 28 March.

[CIT0042] Lovrić, J. (1989b) ‘Bitka za Bosnu’, *Danas*, 25 July.

[CIT0043] Malešević, K. (1991) ‘Marginalije o Be Ha izborima ‘90’, *Revija za sociologiju*, 22, 2.

[CIT0044] Marinković, G. (1990) ‘Rasap komunista Hrvatske: Kako dobiti izbore, a ne izgubiti Srbe’, *Danas*, 20 February.

[CIT0045] Mikulandra, D. (1990) ‘Tuđe nećemo, Bosnu ne damo!’, *Slobodna Dalmacija*, 27 May.

[CIT1001] Rae, D. W. (1995) ‘Using District Magniture to Regulate Political Party Competition', *Journal of Economic Perspectives*, 9, 1.

[CIT0046] Ramet, S. P. (2006) *The Three Yugoslavias: State Building and Legitimation, 1918–2005* (Bloomington, IN, Indiana University Press).

[CIT0047] Remington, T. F. & Smith, S. S. (1996) ‘Political Goals, Institutional Context, and the Choice of an Electoral System: The Russian Parliamentary Election Law’, *American Journal of Political Science*, 40, 4.

[CIT0048] Renwick, A. (2005) ‘Modelling Multiple Goals: Electoral System Preferences in Hungary in 1989’, *Europe-Asia Studies*, 57, 7.

[CIT0049] Ribičič, C. (1995) *Siva tipka 074: Bili su mi dragi* (Zagreb, Birotisak).

[CIT0050] Rogowski, R. (1987) ‘Trade and the Variety of Democratic Institutions’, *International Organization*, 41, 2.

[CIT0051] Rokkan, S. (1970) *Citizens, Elections, Parties: Approaches to the Comparative Study of the Processes of Development* (New York, NY, David McKay).

[CIT0052] Romanić, K. (1989) *Zakon o izborima: Predgovor i objašnjenja* (Belgrade, Službeni list SFRJ).

[CIT0053] Schröder, V. & Manow, P. (2020) ‘An Intra-Party Account of Electoral System Choice’, *Political Science Research and Methods*, 8, 2.

[CIT0054] Šentija, J. (2005) *Ako Hrvatske bude: Zapisi iz onih godina* (Zagreb, Školska knjiga).

[CIT0055] Skupština SRBiH (1989a) ‘Zakon o izborima’, *Službeni list SRBiH*, 45, 1177–88.

[CIT0056] Skupština SRBiH (1989b) ‘Zakon o izbornim jedinicama za izbor delegate u vijeća Skupštine Socijalističke Republike Bosne i Hercegovine i u Savezno Vijeće Skupštine Socijalističke Federativne Republike Jugoslavije’, *Službeni list SRBiH*, 45, 1189–91.

[CIT0057] Skupština SRBiH (1990) ‘Zakon o izboru odbornika i poslanika u skupštine društveno-političkih zajednica’, *Službeni list SRBiH*, 21, 596–605.

[CIT0058] Sokol, S. (1988) ‘Skupštinski sistem i ustavne promjene’, in Gajski, M. (ed.) *Promjene Ustava SFRJ: Prilog javnoj raspravi* (Zagreb, Pravni fakultet).

[CIT0059] Šola, M. (1990) ‘Samo da srce ne prevlada nad razumom’, *Komunist*, 13 April.

[CIT0060] Spehnjak, K. (1991) ‘Funkcioniranje “plebiscitarne demokracije” u Hrvatskoj 1945–1952 (Izborni aspect organizacije legitimacijskog procesa)’, *Časopis za suvremenu povijest*, 23, 1–3.

[CIT0061] Tucker, J. A. (2002) ‘The First Decade of Post-communist Elections and Voting: What Have We Studied and How Have We Studied It?’, *Annual Review of Political Science*, 5.

[CIT0062] Živković, R. & Habul, E. (1990) ‘Bosna neće tutora’, *Oslobođenje*, 26 May.

